# Discovery of a new zinc oxide semiconductor: 21R polytype

**DOI:** 10.1063/4.0000296

**Published:** 2025-03-27

**Authors:** Matej Fonović, Jelena Zagorac, Maria Čebela, Dragana Jordanov, Dejan Zagorac

**Affiliations:** 1 Faculty of Engineering, University of Rijeka, Rijeka, Croatia; 2 Center of Excellence “Center for Synthesis, Processing and Characterization of Materials for Application in Extreme Conditions—Cextreme Lab,” Belgrade, Serbia; 3 Vinča Institute of Nuclear Sciences—National Institute of the Republic of Serbia, University of Belgrade, Belgrade, Serbia

## Abstract

Zinc oxide (ZnO) is a notable semiconductor with a range of interesting electronic and optical properties. Polytypic behavior of crystal structures can strongly affect the properties of materials, especially in ZnO. We report the first prediction of a new 21R polytype in zinc oxide with advanced properties. Ab initio calculations were carried out using two-hybrid functionals: HSE06 and PBE0. Structural properties of different ZnO polytypes were investigated, and theoretical data concurred with experimental results. This can be further exploited for various applications based on their unique properties. Electronic properties were studied using band structures and density of states (DOS). Present DFT calculations agree very well with previous calculations and measurements of known ZnO polytypes, and the new 21R polytype is found as a direct band gap semiconductor. The size of the band gap in the case of the hybrid HSE06 functional is calculated to be 2.79 eV and with PBE0 is 3.42 eV. Understanding the structure–property relationship helps in tailoring ZnO for specific applications and optimizing its performance in various technological contexts, especially as an advanced semiconductor material, with possible applications such as 0D, 1D, 2D, and 3D materials.

## I. INTRODUCTION

Zinc oxide (ZnO) is a versatile compound with a wide range of applications and interesting properties. It is used in various electronic devices such as transistors, diodes, and light-emitting diodes (LEDs). It has applications in photocatalysis, electronics, high-efficiency solar cells, cosmetics, pharmaceuticals, biomaterials, ceramics, and others. The ability of ZnO to exist in different crystal structures allows tuning of its semiconducting properties, which are essential for its applications in optoelectronics, ultraviolet (UV) devices, and sensors.^1–4^ Doping or substituting cations/anions is particularly intriguing for the technological use of ZnO and similar Zn-based materials, as even a minimal addition of another substance can significantly influence their properties (electrochemical,^5,6^ electronic,^7,8^ magnetic,^9,10^ photocatalytic,^11–13^ etc.) and applications (biomedical,^14^ gas sensing,^15^ environmental,^16,17^ etc.).

ZnO, as well as structurally related ZnS, crystallizes in two main crystalline forms, cubic sphalerite and hexagonal wurtzite. Wurtzite (2H, space group *P6_3_mc*) is the most common and stable polytype of ZnO at room temperature. However, the natural form of ZnO is rarely found in its pure form, as the mineral zincite.^1,4^ On the other hand, sphalerite is commonly found in zinc sulfide,^18,19^ while ZnO–ZnS structures are proposed as a new efficient and earth-abundant absorber material for solar cells,^20,21^ core–shell structures,^22,23^ or ZnO_1−x_S_x_ alloys^24–27^ with various industrial and technological applications.

**FIG. 1.**
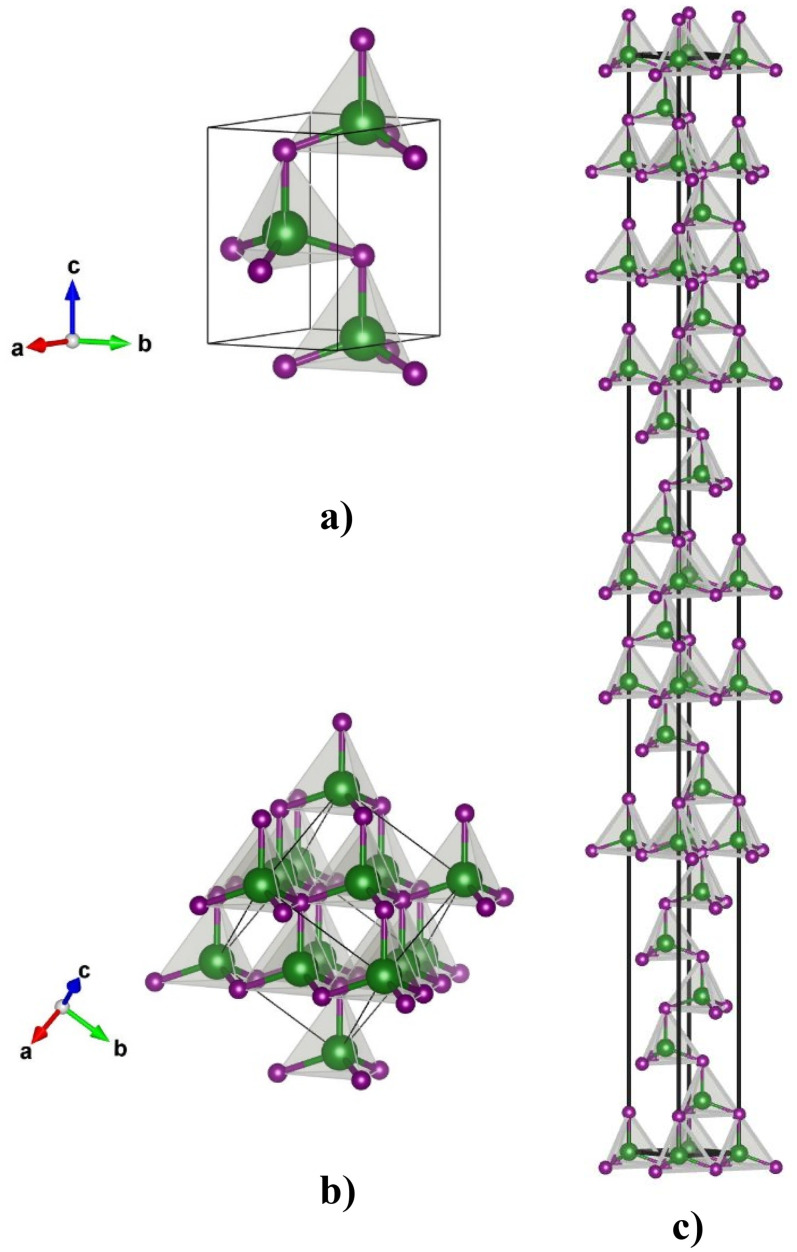
Visualization of the experimentally observed wurtzite (2H) type (a) and sphalerite (3C) type (b) and predicted 21R polytype (c) of zinc oxide. Green and purple spheres denote Zn, and O atoms, respectively.

In ZnO, cubic sphalerite (3C) is a metastable phase appearing in space group *F-43m* and can be achieved in nanocrystalline ZnO thin films by growing ZnO on substrates with cubic lattice structure^28–30^ [Fig. 1(b)]. The transition from sphalerite ZnS to (metastable) wurtzite can occur at a temperature of 1020 °C.^31^ The temperature of the phase transition can be decreased to 350 °C for ZnS nanoparticles (∼5 nm in size), which are in contact with wurtzite-like ZnO nanoparticles.^32^ ZnO film deposition below room temperature down to −240 °C reveals structural disorders caused by the irregular occupation of oxygen tetrahedra forming dioxygen species in zinc oxide.^33–35^ With the increase in pressure in the system, one can obtain the rock salt (NaCl) phase of ZnO.^36–39^ Additional calculated or predicted zinc oxide phases can be found in the literature, for example, the BeO type,^40–43^ the NiAs type,^40,44,45^ the CsCl type,^25,40,46^ the 5–5 type,^40,47–49^ GeP type,^50–52^ the WC type,^40,53^ the BN type,^52,54^ the Au type,^55^ the GaTe type,^55^ the InSb type,^55^ the IrU type,^55^ the Na_2_SiO_3_ type,^55^ and many unknown ZnO bulk modifications^40,55–57^ and ZnO monolayers and nanostructures.^4,58–66^ Perhaps the most intriguing calculations in the ZnO are the prediction of various polytypes.

Polytypism in materials science refers to the existence of different structural variations (or polytypes) within a material that share the same basic chemical composition but differ in their atomic arrangement or crystal structure.^67–72^ For zinc oxide, polytypism is an important concept because it influences the material's physical properties and applications. While there exist about 200 experimentally identified stacking variants of ZnS,^73,74^ ZnO has only three experimentally known bulk phases: wurtzite and sphalerite under ambient conditions and a rock salt phase at high pressures. Previous theoretical research has found several polytypes existing in pristine ZnO as well as in mixed ZnO/ZnS chemical systems from 4H to 15R polytypes.^30,75–78^ Motivation for this study is to find possible higher-order polytype in zinc oxide, which could have advanced properties. In this paper, we predict a new ZnO 21R complex polytype and investigate its crystal structure and electronic properties.

## II. METHODS

Our general approach to crystal structure prediction and determination of structure candidates on the energy landscape of the system of interest is explained elsewhere.^2,40,79^ The *ab initio* calculations were performed using the CRYSTAL17 code, based on the linear combination of atomic orbitals.^80^ The local optimizations of structures employed analytical gradients.^81^ Local optimizations and band structure calculations were performed on the density functional theory (DFT) level, employing the HSE06 and PBE0 hybrid functionals. The hybrid HSE06 (Heyd–Scuseria–Ernzerhof) exchange-correlation functional uses an error-function-screened Coulomb potential to calculate the exchange portion of the energy to improve computational efficiency,^82^ while the PBE0 functional mixes the Perdew–Burke–Ernzerhof (PBE) exchange energy and Hartree–Fock exchange energy in a 3:1 ratio, along with the full PBE correlation energy.^83,84^ Using several different *ab initio* methods is highly useful to get some feeling for the quantitative validity of the results (especially in cases where no experimental data are available for comparison).^78,85,86^ On the other hand, it is very important that our prediction methods are in good agreement with experiments where such data exists.^87–90^ Each calculation employed an all-electron basis set:^91^ a [6s5p2d] basis set in the case of zinc, and a [4s3p] basis set in the case of oxygen.^40,76^ In each structural optimization, Fock/KS matrix mixing was set to 30%, and the tolerances for the convergence on energy were set to 10^−7^. A shrinking factor of 8 × 8 × 8, to generate a commensurate grid of k-points in reciprocal space, according to Monkhorst–Pack scheme has been used. Structural and crystallographic analysis has been performed using the KPLOT package^92^ and visualized using the VESTA code.^93^

## III. RESULTS AND DISCUSSION

### A. Structural properties

Wurtzite has a hexagonal crystal structure characterized by a lattice with alternating *ABAB* layers of zinc and oxygen atoms in a hexagonal *hcp* stacking sequence. The wurtzite polytype is denoted as 2H, where 2 indicates two-layer stacking periodicity and H indicates hexagonal symmetry.^30^ This periodicity doubles, triples, and quadruples in the 4H, 6H, and 8H polytypes, respectively.^94^ The number 3 in the 3C polytype refers to the three-layer periodicity of the *fcc* stacking (*ABC*) and the letter C denotes the cubic symmetry of the crystal [see Fig. 1(b)]. The family of rhombohedral polytypes is labeled with R, for example, 9R, 12R, and 15R.^74,95^ With the increase in the polytype number, the stacking sequences become more complex. In this study, we predict a new complex 21R polytype of zinc oxide as shown in Fig. 1(c). The 21R polytype features a complex stacking sequence where the atomic layers are arranged in a repeating pattern every 21 layers in a rhombohedral unit cell and appear in a structurally related ZnS chemical system.^96^ Computed unit cell parameters (converted to hexagonal lattice) and atomic positions computed with hybrid HSE06 and PBE0 functionals are shown in Table I.

**TABLE I.** Calculated structural details of the newly predicted 21R polytype crystal in ZnO. Local optimizations were performed with the PBE0 and HSE06 hybrid functionals.

Modification, space group	Unit cell parameters and atomic positions	
	HSE06	PBE0
21R polytype *R3m* (no. 160)	*a* = 3.252 Å;	*a* = 3.250 Å;
	*c* = 55.171 Å	*c* = 55.143 Å
	Zn1 0 0 0	Zn1 0 0 0.9996
	Zn2 0 0 0.7144	Zn2 0 0 0.7140
	Zn3 0 0 0.4290	Zn3 0 0 0.4286
	Zn4 0 0 0.8099	Zn4 0 0 0.8095
	Zn5 0 0 0.5239	Zn5 0 0 0.5235
	Zn6 0 0 0.9047	Zn6 0 0 0.9043
	Zn7 0 0 0.2859	Zn7 0 0 0.2855
	O1 0 0 0.0362	O1 0 0 0.0358
	O2 0 0 0.7509	O2 0 0 0.7505
	O3 0 0 0.4652	O3 0 0 0.4648
	O4 0 0 0.8459	O4 0 0 0.8455
	O5 0 0 0.5599	O5 0 0 0.5596
	O6 0 0 0.9411	O6 0 0 0.9407
	O7 0 0 0.3219	O7 0 0 0.3215

A summary of experimentally observed and theoretically predicted and calculated ZnO structures is shown in Table II. We note that present calculations of wurtzite (2H) modification using hybrid HSE06 (a = 3.266 Å, c = 5.202 Å) and PBE0 (a = 3.264 Å, c = 5.199 Å) functionals are in excellent agreement with previous calculations^40,76,78,97,98^ and synthesized 2H structures.^99–101^ The internal parameter *u* for the wurtzite 2H is computed to *u* = 0.3826, regardless of computational approach, and concur with measured data for *u* = 0.3820–0.3990.^99–101^ Similarly, calculated unit cell parameters for sphalerite (3C) structure using hybrid HSE06 (a = 4.581 Å) and PBE0 (a = 4.579 Å) functionals are in excellent agreement with previous calculations^40,76,78,97,98^ and with experimental data on 3C structure.^29,102^ On the one hand, there is much experimental evidence of the hexagonal wurtzite modification, while on the other hand, the cubic sphalerite phase is not easy to synthesize and various predicted polytypes of ZnO have not been experimentally observed so far. The novel 21R polytype is the first model to be predicted in a zinc oxide chemical system. Therefore, it is not possible to directly compare present structural data; however, present calculations using hybrid approximations concur with previously predicted and calculated ZnO polytypes, indicating the feasibility of the predicted 21R ZnO polytype.

**TABLE II.** A summary of experimentally observed and theoretically predicted and calculated unit cell parameters of the ZnO polytypes, and experimental results when existing. Cell parameters are given in angstrom (Å).

Structure type Space group		Calculated and measured unit cell parameters (Å)					
	*HSE06*	PBE0	LDA	*B3LYP*	HF	Theory (other)	Experiment
Wurtzite (2H)	*a* = 3.266	*a* = 3.264	*a* = 3.19,	*a* = 3.28	*a* = 3.29	a = 3.281	*a* = 3.25
*P*6_3_*mc* (no. 186)	*c* = 5.202^a^	*c* = 5.199^a^	*c* = 5.18^c^^,^^d^	*c* = 5.29^c^^,^^d^	*c* = 5.24^c^^,^^d^	c = 5.267^e^	*c* = 5.21^g^
	*a* = 3.27	*a* = 3.26				a = 3.283	a = 3.258
	*c* = 5.20^b^	*c* = 5.20^b^				c = 5.309^f^	c = 5.220^h^
Sphalerite (3C)	*a* = 4.581^a^	*a* = 4.579^a^	*a* = 4.49^c^^,^^d^	*a* = 4.61^c^^,^^d^	*a* = 4.62^c^^,^^d^	a = 4.634^e^	*a* = 4.595^i^
*F-43m* (no. 216)	*a* = 4.58^b^	*a* = 4.58^b^				a = 4.627^f^	a = 4.63^j^
4H	*a* = 3.25	*a* = 3.25	*a* = 3.19	*a* = 3.28	*a* = 3.28	*a* = 3.278	n/a
*P6_3_mc* (no. 186)	*c* = 10.51^b^	*c* = 10.50^b^	*c* = 10.38^c^	*c* = 10.64^c^	*c* = 10.60^c^	*c* = 10.650^k^	
						*a* = 3.216	
						*c* = 10.433^l^	
5H	*a* = 3.25	*a* = 3.25,	*a* = 3.19	*a* = 3.27	*a* = 3.27	n/a	n/a
*P3m1* (no. 156)	*c* = 13.15^b^	*c* = 13.14^b^	*c* = 12.95^c^	*c* = 13.29^c^	*c* = 13.25^c^		
6H	*a* = 3.25	*a* = 3.25	*a* = 3.18	*a* = 3.27	*a* = 3.27	*a* = 3.275	n/a
*P6_3_mc* (no. 186)	*c* = 15.80^b^	*c* = 15.80^b^	*c* = 15.57^c^	*c* = 15.97^c^	*c* = 15.92^c^	*c* = 15.989^k^	
						*a* = 3.200	
						*c* = 15.894^l^	
8H	*a* = 3.24	*a* = 3.24	*a* = 3.18	*a* = 3.27	*a* = 3.27	n/a	n/a
*P6_3_mc* (no. 186)	*c* = 21.10^b^	*c* = 21.09^b^	*c* = 20.78^c^	*c* = 21.30^c^	*c* = 21.27^c^		
9R	*a* = 3.26	*a* = 3.25	*a* = 3.19	*a* = 3.28	*a* = 3.28	n/a	n/a
*R3mH* (no. 160)	*c* = 23.57^b^	*c* = 23.56^b^	*c* = 23.42^c^	*c* = 23.98^c^	*c* = 23.74^c^		
12R	*a* = 3.25	*a* = 3.25	*a* = 3.19	*a* = 3.27	*a* = 3.28	*a* = 3.269	n/a
*R3mH* (no. 160)	*c* = 31.51^b^	*c* = 31.50^b^	*c* = 31.28^c^	*c* = 32.04^c^	*c* = 31.75^c^	*c* = 31.904^m^	
15R	*a* = 3.25	*a* = 3.25	*a* = 3.28	*a* = 3.15	*a* = 3.28	n/a	n/a
*R3mH* (no. 160)	*c* = 39.46^b^	*c* = 39.44^b^	*c* = 39.77^c^	*c* = 38.32^c^	*c* = 39.77^c^		
21R	*a* = 3.252	*a* = 3.250	n/a	n/a	n/a	n/a	n/a
*R3mH* (no. 160)	*c* = 55.171^a^	*c* = 55.143^a^					

^a^ Present study.

^b^ Ref. 78.

^c^ Ref. 76.

^d^ Ref. 40.

^e^ Ref. 97.

^f^ Ref. 98.

^g^ Refs. 99 and 100.

^h^ Ref. 101.

^i^ Ref. 29.

^j^ Ref. 102.

^k^ Ref. 77.

^l^ Ref. 103.

^m^ Ref. 55.

### B. Electronic properties

Zinc oxide is a known semiconductor and shows a direct and wide band gap of around 3.3–3.4 eV, which has been measured and computed for the equilibrium wurtzite phase.^1,2^ The electronic structure of ZnO can experimentally be tuned by using cubic sphalerite or high-pressure rock salt modifications.^78^ Moreover, there exists theoretical data for band gap tuning using various ZnO modifications, including GeP, 5–5, NiAs, BeO, and CsCl.^40,97^ However, the most intriguing effect is found in the ZnO polytypes where different polytypes (with/or without additional sulfur doping) can dramatically affect electronic properties, especially direct–indirect band gap character and discovery of the so-called secondary band gap.^30,75–78^

**FIG. 2.**
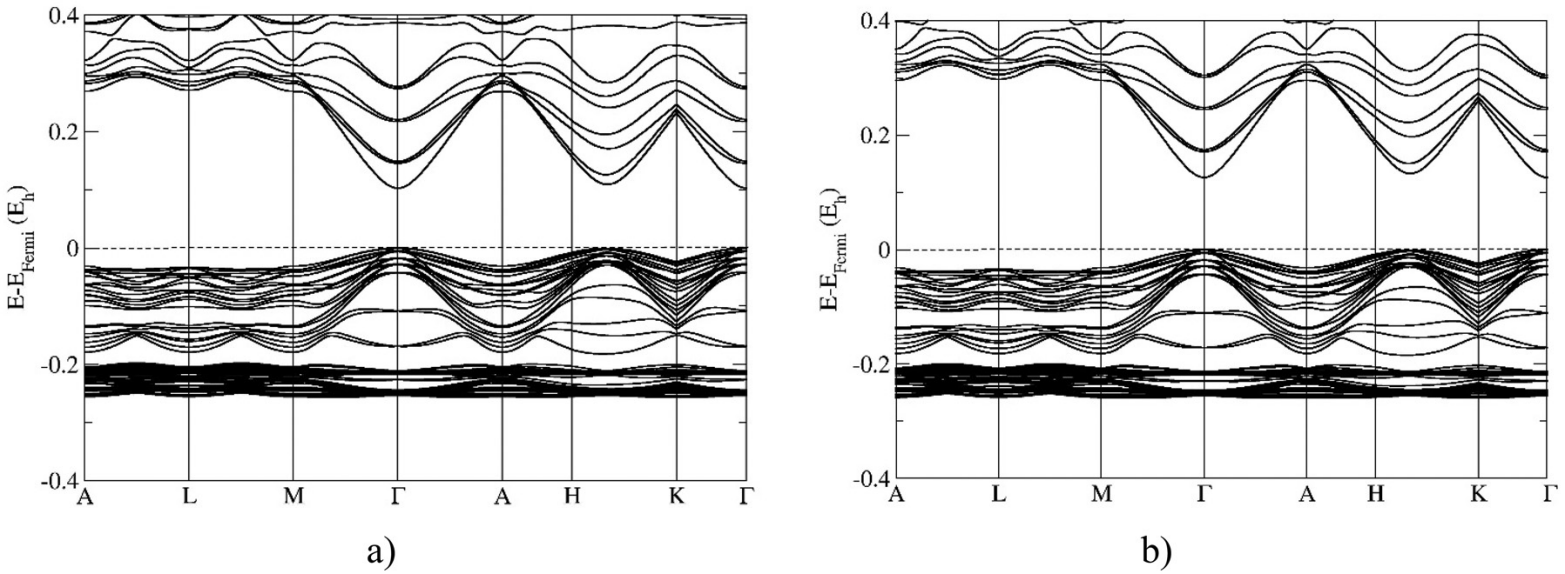
Band structures of the 21R polytype in zinc oxide computed using: (a) the HSE06 and (b) the PBE0 functional. Note that the labels of the special points of the Brillouin zones correspond to those of a hexagonal lattice.

The band structure of the newly predicted 21R polytype of ZnO calculated using various hybrid functionals is presented in Fig. 2. The size of the bandgap in the case of the hybrid HSE06 functional is calculated to be 2.79 eV and with PBE0 is 3.42 eV. This is in very good agreement with previous theoretical calculations on rhombohedral polytypes 9R, 12R, and 15R which band gaps were computed with HSE06 in the range 2.77–2.82 eV, and with PBE0 functional in the range 3.40–3.45 eV.^78^ Band structure calculations show a direct bandgap at the Γ point of the Brillouin zone regardless of the computational approach [Figs. 2(a) and 2(b)]. The top of the valence band (TVB) and the bottom of the conduction band (BCB) along the H–K direction of the Brillouin zone are narrowed suggesting the possible development of a so-called secondary gap, observed also in previously computed ZnO polytypes, especially in rhombohedral polytypes (such as 9R, 12R, and 15R).^76,78^ Such complex polytypic changes in the structure of zinc oxide can lead to a dramatic restructuring of electronic structure, especially of direct–indirect band gap transition region, thus leading to modification of their electronic, optical, and physical properties.

**FIG. 3.**
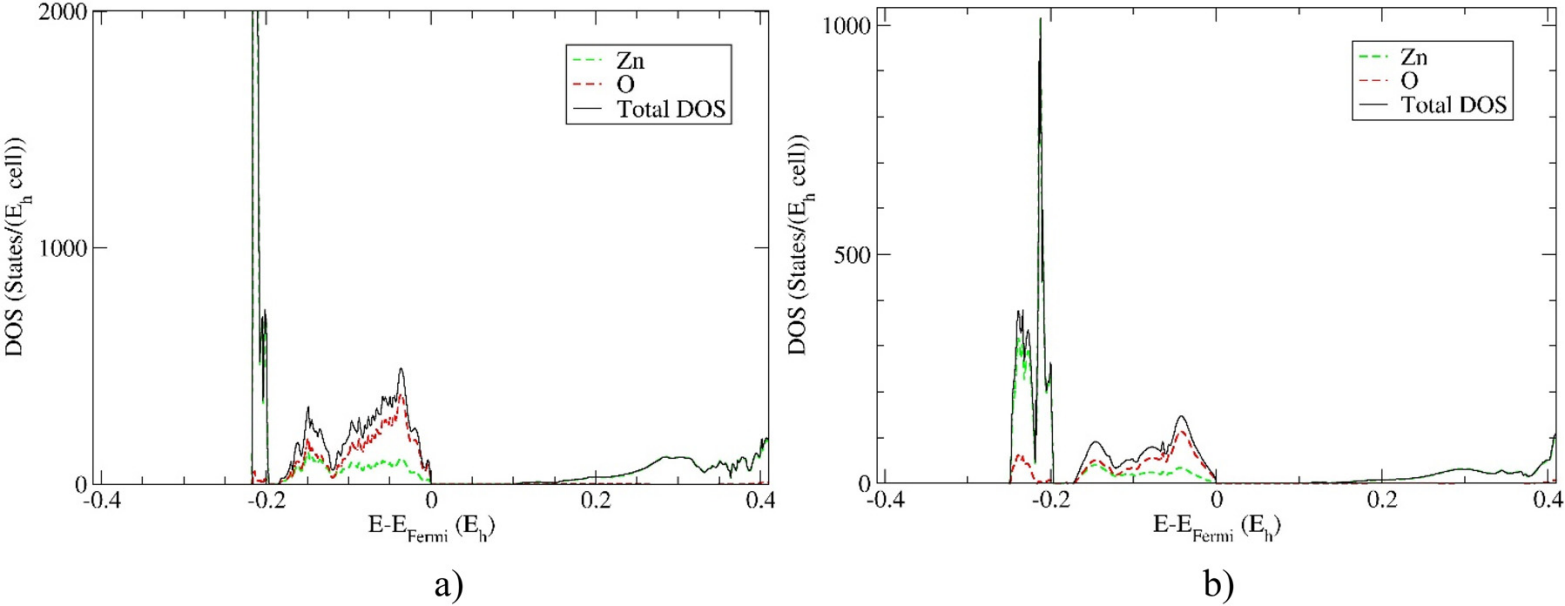
The density of states (DOS) of the: (a) 21R polytype and (b) wurtzite (2H) modification. Calculations were accomplished using hybrid HSE06 functional.

The DOS calculations of the 21R polytype and wurtzite (2H) modification computed using hybrid HSE06 functional are shown in Fig. 3. The predicted 21R polytype shows similar electronic properties as experimentally observed 2H modification, where the direct bandgap is observed at the Γ-point and where O atoms mostly influence the TVB. Interestingly, huge peaks caused by Zn atom bands are clearly visible in the DOS in the energy range of −0.2 Hartree below the TVB. The Zn atoms are dominating the BCB as previously found in calculations of wurtzite modification.^40,77,98,103,104^

**FIG. 4.**
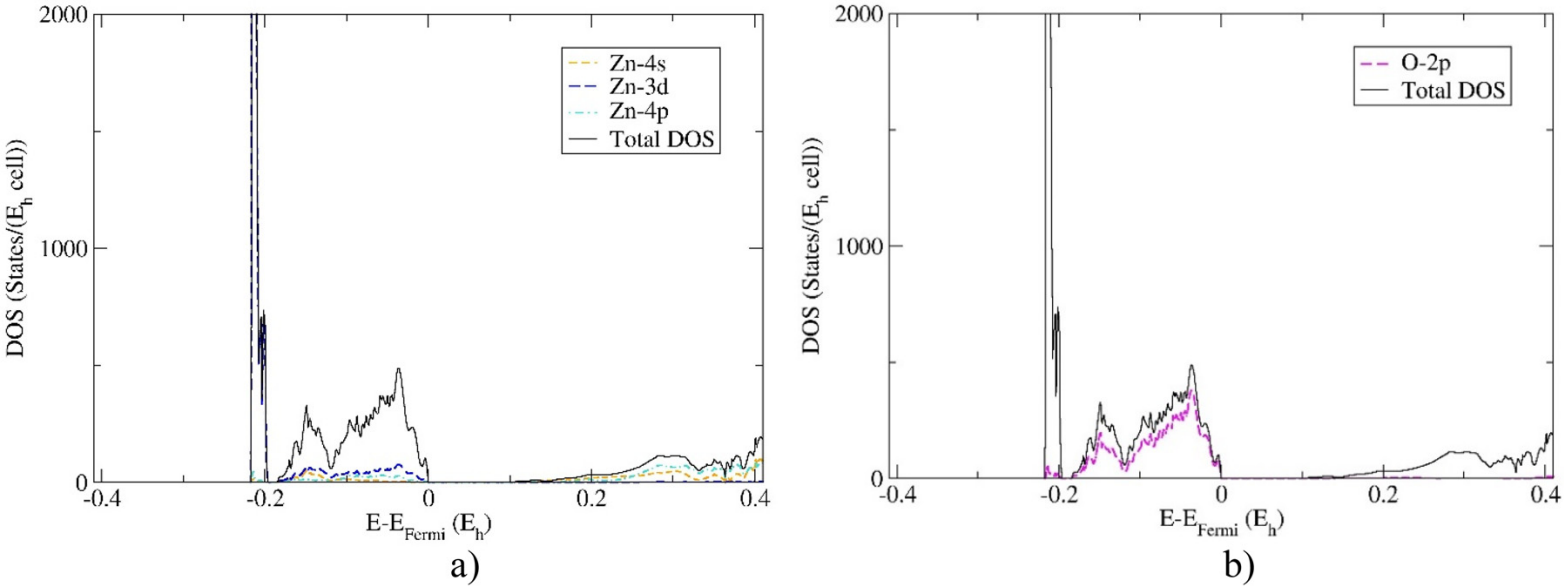
Partial DOS projected: (a) on the Zn -*4s, -3d,* and -*4p* atomic orbitals (AO) and (b) on the O -*2p* AO of the 21R polytype ZnO modification. Calculations were performed using hybrid HSE06 functional.

**FIG. 5.**
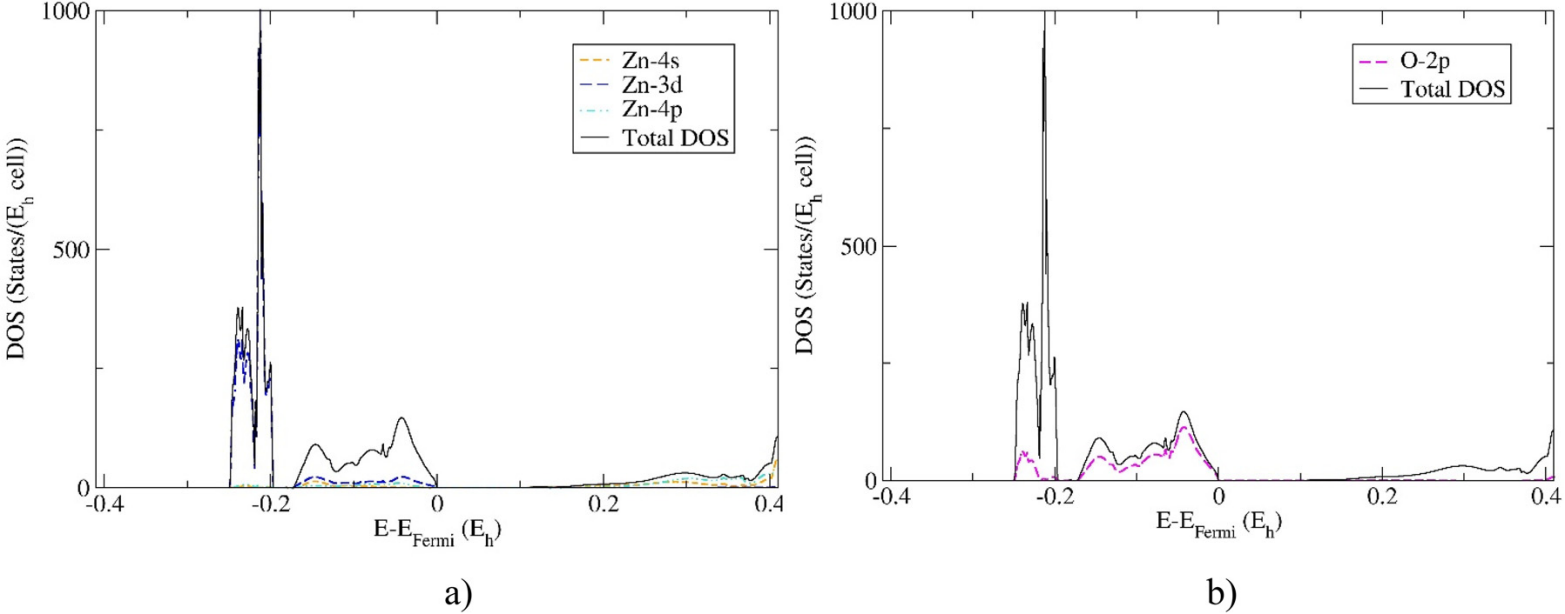
Partial DOS projected: (a) on the Zn *-4s, -3d,* and -*4p* AO and (b) on the O -*2p* AO of the wurtzite 2H ZnO modification. Calculations completed using hybrid HSE06 functional.

Furthermore, the partial DOS calculations projected on the Zn and O atomic orbitals of the 21R polytype and 2H ZnO modification are shown in Figs. 4 and 5. It has been found that huge peaks are caused by Zn *3d* electrons in both modifications. The TVB is dominated by oxygen *2p* electrons, while BCB is first dominated by *4s* followed by *4p* electronic orbitals. These results confirm previous experimental and theoretical investigations for wurtzite structure,^40,77,98,103,104^ which is a strong indication for reliable prediction of electronic properties of novel 21R polytype.

Finally, a summary of calculated and measured band gaps of different polytype structures in ZnO is presented in Table III. Wurtzite 2H is computed to have 2.89 and 3.53 eV using the HSE06 and PBE0 methods, respectively. Similarly, 3C modification is calculated to have 2.70 and 3.33 eV using the same hybrid functionals. Calculated bandgap values for 2H and 3C polytypes concur with previous calculations and measurements.^24,29,78,97,104–119^ Since the new 21R polytype is the first predicted model in a zinc oxide it is not possible to compare computed bandgap energies, but present calculations using hybrid functionals agree very well with previously calculated band gaps for lower order ZnO polytypes (Table III).^78,108^

**TABLE III.** A summary of calculated and measured band gaps of different polytype structures in ZnO. Band gap energies are shown in electronvolt (eV).

Structure type	Calculated and measured band gap energies (eV)				
	HSE06	PBE0	B3LYP	Theoretical (other)	Experimental
Wurtzite (2H)	2.89^a^ 2.89^b^	3.53^a^ 3.53^b^	3.21^b^	2.901^c^ 2.68^d^ 2.65^e^ 3.3^f^ 3.479^g^	3.44^h^ 3.40^i^ 3.25^j^ 3.20^k^ 3.30^l^
Sphalerite (3C)	2.70^a^ 2.70^b^	3.33^a^ 3.33^b^	3.08^b^	2.679^c^ 3.381^g^ 3.225^m^ 3.30^n^ 3.18^f^ 2.50^e^	3.28^o^ 3.22^p^
4H	2.79^b^	3.42^b^	3.15^b^	3.425^g^	n/a
5H	2.76^b^	3.39^b^	3.12^b^	n/a	n/a
6H	2.76^b^	3.39^b^	3.11^b^	3.410^g^	n/a
8H	2.74^b^	3.37^b^	3.09^b^	n/a	n/a
9R	2.82^b^	3.45^b^	3.16^b^	n/a	n/a
12R	2.78^b^	3.42^b^	3.08^b^	n/a	n/a
15R	2.77^b^	3.40^b^	3.13^b^	n/a	n/a
21R	2.70^b^	3.33^b^	n/a	n/a	n/a

^a^ Present study.

^b^ Ref. 78.

^c^ Ref. 97.

^d^ Ref. 105.

^e^ Refs. 24 and 106.

^f^ Refs. 104 and 107.

^g^ Ref. 108.

^h^ Ref. 109.

^i^ Refs. 110–112.

^j^ Ref. 113.

^k^ Refs. 114 and 115.

^l^ Ref. 116.

^m^ Ref. 117.

^n^ Ref. 118.

^o^ Ref. 29.

^p^ Ref. 119.

## IV. CONCLUSION

Polytypism in ZnO illustrates the material's ability to adopt different crystal structures, each with distinct properties and potential applications. Understanding and controlling these polytypes is essential for optimizing ZnO for various technologies, from optoelectronics to sensors and high-frequency devices. Here, we report the crystal structure of a new 21R polytype in ZnO and the structure–property relationship. *Ab initio* calculations were performed using HSE06 and PBE0 hybrid functionals. The 21R polytype features a complex stacking sequence and appears structurally related to zinc sulfide. Known 2H and 3C polytypes, as well as newly predicted 21R polytype, were compared to the experimentally observed and previously calculated ZnO structures. Present calculations of wurtzite (2H) and sphalerite (3C) modifications using hybrid HSE06 and PBE0 functionals are in excellent agreement with structural data from previous calculations and synthesized 2H and 3C structures. Similarly, calculated band gap values for 2H and 3C polytypes concur with previous calculations and measurements. The size of the band gap was calculated E_gap_ = 2.79 eV (HSE) and E_gap_ = 3.42 eV (PBE0), which was in very good agreement with previous theoretical calculations on rhombohedral polytypes 9R, 12R, and 15R. Band structure calculations show a direct band gap at the Γ point of the Brillouin zone regardless of the computational approach in agreement with the literature. The discovery of a possible secondary band gap might influence direct–indirect band gap character, which is highly important for optoelectronic applications, while the discovery of a 21R polytype could be used as a template for versatile ZnO materials and heterostructures.

## Data Availability

The data that support the findings of this study are available from the corresponding authors upon reasonable request.
